# Genotoxic Effects of Semi-Synthetic Isodillapiole on Oviposition in *Aedes aegypti* (Linnaeus, 1762) (Diptera: Culicidae)

**DOI:** 10.1590/0037-8682-0467-2020

**Published:** 2020-12-11

**Authors:** Luiz Henrique Fonseca dos Santos, Pedro Rauel Cândido Domingos, Sabrina da Fonseca Meireles, Leticia Cegatti Bridi, Ana Cristina da Silva Pinto, Míriam Silva Rafael

**Affiliations:** 1 Instituto Nacional de Pesquisas da Amazônia, Programa de Iniciação Científica, Manaus, AM, Brasil.; 2 Faculdade Uninassau de Manaus, Setor Saúde, Manaus, AM, Brasil.; 3 Instituto Nacional de Pesquisas da Amazônia, Programa de Pós-Graduação em Genética, Conservação e Biologia Evolutiva, Manaus, AM, Brasil.; 4 Universidade Federal do Amazonas, Programa de Pós-Graduação em Biotecnologia, Manaus, AM, Brasil.; 5 Instituto Nacional de Pesquisas da Amazônia, Coordenação de Sociedade, Ambiente e Saúde, Manaus, AM, Brasil.

**Keywords:** Biological control, Dillapiole, Nuclear abnormality, Micronuclei

## Abstract

**INTRODUCTION:**

Semi-synthetic dillapiole compounds derived from *Piper aduncum* essential oil are used as alternative insecticides to control insecticide-resistant *Aedes aegypti*. Thus, we aimed to evaluate the genotoxic effects of semi-synthetic isodillapiole on the nuclei of neuroblasts (larvae) and oocytes (females) and the mean oviposition rates of the females over four generations (G_1_, G_2_, G_3_, and G_4_) of *Ae. aegypti*.

**METHODS:**

Larvae were captured in the city of Manaus, Amazonas state, Brazil, and exposed to isodillapiole in bioassays (20, 40, and 60 µg/mL) and a negative control (0.05% DMSO in tap water) for 4 h. The cerebral ganglia were extracted from the larvae and oocytes from the adult females to prepare slides for cytogenetic analysis. Breeding pairs were established and eggs counts were quantified taken after the bioassays.

**RESULTS:**

The analysis of 20,000 interphase nuclei of neuroblasts and oocytes indicated significant genotoxicity (micronuclei, budding, polynucleated cells, and other malformations) compared to that of the control. Metaphasic and anaphasic nuclei presented chromosomal breaks; however, no significant variation and damage was observed in the negative control. A significant reduction in mean oviposition rates was also recorded following exposure to isodillapiole over the four generations (G_1_, G_2_, G_3_, and G_4_).

**CONCLUSIONS:**

The toxic and genotoxic effects of isodillapiole on *Ae. aegypti* were caused by reduced oviposition in the females and nuclear abnormalities over the four generations of the trials. Further studies are required, rather than our *in vitro* assays, to verify the efficacy of exposure to this compound for controlling *Ae. aegypti.*

## INTRODUCTION

*Aedes aegypti* transmit the four dengue virus serotypes (DENV-1, DENV-2, DENV-3, and DENV-4), chikungunya, urban yellow fever, and the Zika virus^1^ ^,^ ^2^ ^,^ ^3^ ^,^ ^4^. Of these, dengue fever has had the greatest impact on human populations in recent decades. In Brazil, 264,262 probable cases of dengue fever were reported in 2018, while in the USA, a total of 560,586 cases were reported^5^. 

In recent decades, chemical insecticides, in particular temephos and deltamethrin, have been used in programs for the control of *Aedes* mosquitos, such as Brazilian National Dengue Fever Control Program (PNCD)^6^ . These authors argue that it has resulted in the development of resistance in these insects, which may be related to a reduction in either the penetration rate of the insecticide or its metabolism by the insect^6^. This has led to increasing interest and research into the potential of essential oils and other compounds derived from plants as alternatives to insecticides for the control of mosquito disease vectors^7^ ^,^ ^8^ ^,^ ^9^. 

Dillapiole is derived from the essential oil of the spiked pepper plant *Piper aduncum* (Piperaceae), which has potential as a bioinsecticide for the control of insect pests^10^. This compound, which is abundant in the essential oil of *P. aduncum*, is a phenyl ether, and functions as a fungicide, bactericide, and molluscicide^11^ ^,^ ^12^.

The semi-synthetic derivatives of dillapiole are isodillapiole, methyl ethers, ethyl, propyl, butyl, and octil dillapiole, and dillapiole epoxide, and these derivatives have adulticide effects in *Ae. aegypti* ^13^. The variation in the activity of these compounds is related to the differences in the dillapiole side chain, which directly influences its larvicidal effects in *Ae. aegypti*, *Anopheles darlingi*, and *Culex quinquefasciatus* ^13^. 

Dillapiole has toxic and genotoxic effects in the larvae and adult mosquitos of *Ae. aegypti* ^8^ ^,^ ^13^ ^,^ ^14^and *Ae. albopictus* ^15^. The karyotype of *Ae. aegypti* has two pairs of autosomes and one pair of sex chromosomes with a diploid number of 2n = 6^8^ ^,^ ^16^.

The scenario of disease transmission by *Ae. aegypti* has changed in recent decades due to the emergence and reemergence of urban transmission cycles of different arboviruses by this invasive mosquito. In *Ae. aegypti* exposed to semi-synthetic derivatives of dillapiole at low concentrations, DNA damage and decreased fertility were observed over four successive generations, with further trials being hampered by the difficulty of obtaining more generations of mosquitoes due to their infertility following exposure to toxic compounds^8^ ^,^ ^14^. We evaluated the genotoxic effects of isodillapiole in the brain ganglia and oocytes of *Ae. aegypti* to analyze possible chromosomal abnormalities to determine the frequency of changes associated with the decreased fertility of mosquitoes and their descendants over four generations, as well as the potential of this compound as an alternative tool to control *Ae. aegypti*.

## METHODS

### Production of semi-synthetic isodillapiole Flash Column Chromatography

Isodillapiole, a semi-synthetic derivative of dillapiole, was obtained by isomerization with a 17% solution of KOH in ethanol under reflux for 24 h. Isodillapiole was purified in flash Column Chromatography (CC) by elution with hexane; AcOEt (95:5) and MeOH^17^. The mixing of E and Z isomers, based on nuclear magnetic resonance spectroscopy^13^, was conducted at the Laboratory for Research in Natural Products (CPPN) of the National Institute of Amazonian Research (INPA) in Manaus, Amazonas state, Brazil.

### Capture of *Ae. aegypti*

Larvae of *Ae. aegypti* were collected during the Amazonian rainy season from January to March, 2019, both inside and surrounding homes in the Centro neighborhood of the city of Manaus, the capital of the Brazilian state of Amazonas (03°08’33.5″” S, 60°01’13.5”″ W). Collection points were established based on the observed occurrence of *Ae. aegypti* in the area. The larvae were collected in 24 traps made of dark containers placed on wooden pallets (25.4 mm in width × 152.4 mm in length) containing a bait solution of 10% Guinea grass (*Panicum maximum*) in tap water, and these containers were installed in the back gardens of local houses over one week. The pallets and larvae were transported in polypropylene boxes (440 mm in width × 253 mm in height × 355 mm in length) to the Insectarium of the Laboratory of Malaria and Dengue Vectors at the INPA Coordination of Society, Environment, and Public Health (COSAS) in Manaus, Amazonas state. 

The collection of specimens was authorized by the Chico Mendes Institute for Biodiversity Conservation (ICMBio) and the Biodiversity Information and Authorization System (SISBIO), through the permanent license number: 32941 (May 21, 2012) for the collection of zoological material, issued to Dr. Míriam Silva Rafael. 

### Establishment of G_1_ colony of *Ae. aegypti*

The larvae captured in the field were raised at 26°C with a relative humidity of 70% and a standard 12 h-12 h light-dark cycle. The larvae were provisioned twice a day with commercial fish food (*Tetra cichlid*) stored in tap water in enamel trays (20 cm × 7 cm). The water and fish food were changed three times a week, following the standard protocol of the insectarium. Once the adults emerged, they were identified using the taxonomic key^18^.

Pairs of adult mosquitos (n = 400) were maintained for 15 days in cages adapted for mating and oviposition. The larvae emerging from the resulting eggs were denominated as G_1_. Adult females (n = 200) from colony G_1_ were fed hamster (*Mesocricetus aureatus*) blood, and the males (n = 200) were fed 10% sugar solution in 40 × 40 cm^2^ screened cages. This setup ensured the production of maximum number of eggs, under authorization number 020/2017 of the Ethics Committee on the Use of Animals (CEUA) / INPA Central Bioterium. 

### Bioassays of *Ae. aegypti* larvae and adults

Third-stage *Ae. aegypti* larvae of the G_1_ colony (n = 200) were exposed to three different isodillapiole treatments (20, 40, and 60 µg/mL), which were diluted in 20 mL distilled water. The choice to use isodillapiole concentrations was based on the LC50 (minimum inhibitory concentration) value^14^, during our toxicity assay, which were necessary to guarantee the survival of the immature insect for the genotoxicity bioassays. The third-stage larvae were divided into five replicates, with 40 larvae in each concentration as well as a negative control of 0.05% DMSO dissolved in tap water. The larvae were exposed for 4 h in all cases. Following the bioassay, ten third-stage larvae from each concentration and the negative control were used for cytological preparations (mitotic chromosomes). The surviving larvae were transferred to enamel containers with water and fish feed for development until the adult phase. Ten adult females from each group were used to prepare the slides for the retrieval of meiotic chromosomes from the ovaries. The surviving mosquito pairs (n = 10) were transferred to cages adapted for mating and oviposition. All procedural steps were repeated for each of the three subsequent generations (G_2_, G_3_, and G_4_). 

### Cytological preparations

A total of 320 specimens of cerebral ganglia of third stage larvae (n = 160) and ovaries of adult females (n = 160) from the bioassays of the four generations (G_1_, G_2_, G_3_, and G_4_) were used for cytological preparations. Cerebral ganglia (mitotic chromosomes) of the third-stage larvae and ovaries (meiotic chromosomes) of the adult females were extracted using a micro-stylus and tweezers and smeared onto glass slides^19^ ^,^ ^20^.

### Genotoxic analysis

Genotoxicity of the isodillapiole in *Ae. aegypti* was evaluated from the relative frequency (%) of nuclear anomalies (interphase and metaphase) in neuroblasts and oocytes. In the bioassays, 20,000 cells were evaluated from each generation (G_1_, G_2_, G_3_, and G_4_), including 5,000 neuroblasts and oocytes each per treatment (20, 40, and 60 µg of isodillapiole) and negative control.

The abnormalities abnormal found in the mitotic and meiotic cells were counted using a mechanical DigiTimer blood cell counter^ADAmTM-CelT^ (SATRA Technology Centre, Telford Way, Kettering, Northamptonshire, UK) and the microphotographs were obtained using an AxioCam MRcA camera under an Axioplan Zeiss light microscope (100× immersion objective with 1×, 1.25×, and 1.6× optovar; Carl Zeiss MicroImaging, Inc., Thornwood, NY, U.S.A.). 

### Mean oviposition per mosquito pair

The eggs obtained from the surviving females (bioassay G_1_) were counted and transferred to polystyrene cups, containing 20 mL distilled water. The eggs hatched and the next generation (G_2_) of mosquitoes was established. These larvae were raised under the standard insectarium conditions (temperature, humidity, light-dark cycle, and provisioning) described previously for the establishment of generations G_3_ and G_4_. The number of eggs produced by each pair of mosquitoes (ten pairs per treatment) was used to calculate the mean and standard deviation of oviposition after each generation. 

### Statistical analysis

The relative frequencies observed in the assessment of genotoxicity (budding, micronuclei, malformations, and chromosomal breaks) and the mean oviposition were evaluated using two-way analysis of variance (ANOVA) (*p* < 0.05), using the isodillapiole concentrations 20, 40, and 60 μg/mL and the generation exposed to each treatment (G_1_ to G_4_) as the variables. Tukey's test (*p* < 0.05) was used to verify variations between pairs of treatments as well as between each treatment and the negative control. The assumption of the normal distribution of the data was assessed *a priori* using the D'Agostino & Pearson and Kolmogorov-Smirnov tests (*p* < 0.05). Statistical analyses were performed using GraphPad Prism software version 6.00.

## RESULTS

**FIGURE 1:**
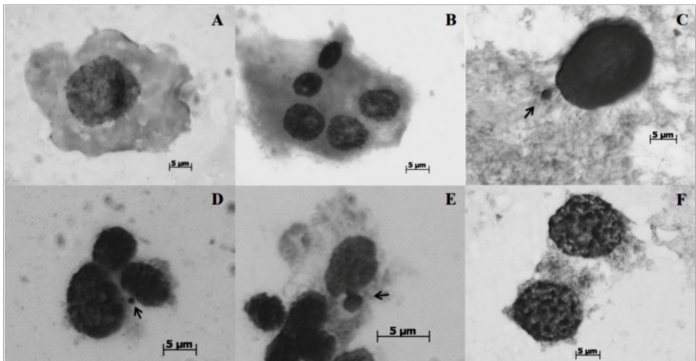
Oocytes (A, B, C, and F) and neuroblasts (D and E) of *Aedes aegypti* treated with isodillapiole over four consecutive generations. A- normal interphase nucleus (negative control group); B- polynucleated cell (after treatment with 20 µg/mL of isodillapiole); C and D- micronucleus (arrow) (60 µg/mL); E- budding (arrow) (40 µg/mL); F- cells in apoptosis (40 µg/mL). Cytological preparations stained with Giemsa (pH 5.8) and orcein-lactic-acetic acid (2%). Magnification: 1000× and 1600×.

**FIGURE 2:**
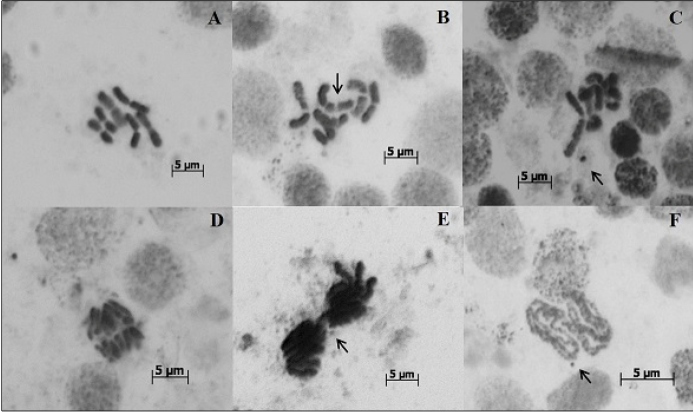
Neuroblasts (A-F) of *Aedes aegypti* treated with isodillapiole over the four consecutive generations (G_1_ to G_4_). A- normal metaphasic chromosomes (in the negative control group). B- chromosome break (arrow) in a metaphasic chromosome; C- fragment of a metaphasic chromosome (arrow); D- normal anaphase (in the negative control group); E- anaphase bridge (arrow); F- fragment of a prometaphasic chromosome (arrow). Cytological preparations stained with Giemsa (pH 5.8) and orcein-lactic-acetic acid (2%). Magnification: 1000× and 1600×.

Cytological preparations of the treated (20, 40, and 60 µg/mL of isodillapiole) cerebral ganglia of the third-stage *Ae. aegypti* larvae and negative controls revealed several abnormalities, including micronuclei, polynucleated cells, budding, and other malformations of the interphase nuclei (Figure 1). Malformations were observed in the metaphasic chromosomes of both the neuroblasts and oocytes, including abnormalities of the chromosomal breakage type, nuclear and anaphase bridges (Figure 2), and cells in apoptosis. 

**FIGURE 3:**
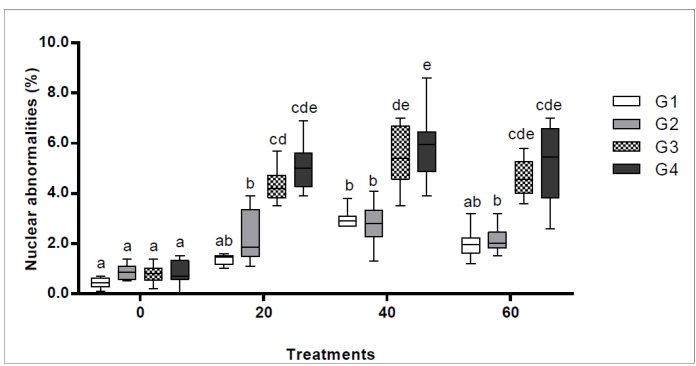
Frequency of nuclear abnormalities (%) in *Aedes aegypti* neuroblast cells observed in the interphase over four consecutive generations (G_1_ to G_4_) monitored in the present study. Different lowercase letters (a-e) indicate a significant difference between the respective treatments (20, 40, or 60 µg/mL of isodillapiole) and the negative control (0 µg/mL), based on Tukey’s test (*p* < 0.05).

The frequency of nuclear abnormalities in the *Ae. aegypti* neuroblasts varied significantly among the three treatment groups (20, 40, and 60 μg/mL of isodillapiole), with frequencies 3.1-6.7 times higher than the control in the first generation and 6.1-7.0 times higher in the fourth generation (ANOVA, *p* < 0.001). In the neuroblasts (Figure 3), compared to the control, chromosomal abnormalities were significantly more frequent in the 20 μg/mL treatment (fourth generation) (Tukey, *p* = 0.01) and 40 μg/mL treatment (third and fourth generations) groups (Tukey, *p* < 0.001).

**FIGURE 4:**
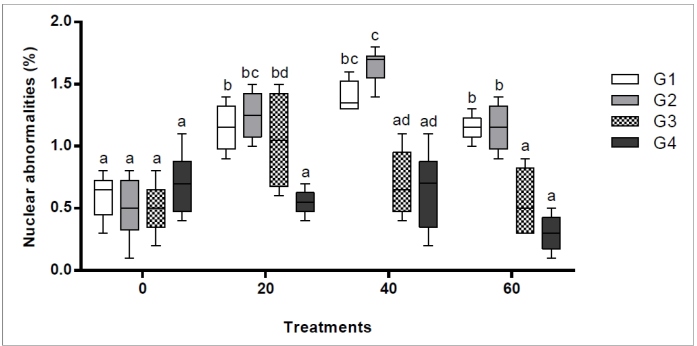
Frequency of nuclear abnormalities (%) observed in *Aedes aegypti* interphase oocyte cells over the four consecutive generations (G_1_ to G_4_). Different lowercase letters (a-b) indicate a significant difference between the respective treatments (20, 40, or 60 µg/mL of isodillapiole) and the negative control (0 µg/mL) based on Tukey’s test (*p* < 0.05).

In the oocytes, the frequency of nuclear anomalies increased from the first to the second generation in all treatment groups (ANOVA, *p* < 0.001). Nevertheless, it decreased in subsequent generations and the negative control and 20 µg/mL isodillapiole treatment in the fourth generation (Tukey, *p* = 0.637), the 40 µg/mL treatment in the third and fourth generations (Tukey, *p* = 0.394 and 0.979, respectively), and 60 µg/mL treatment in the third generation groups (Tukey, *p* = 0.979) did not differ significantly. However, the number of oocytes decreased from G3 to G4 in the oocytes there was a decrease between generations G_3_ and G_4_ due to the high frequency of cells undergoing apoptosis (Figure 4).

No chromosomal damage was found in the cells of the negative control groups in any of the four generations. However, the chromosomal damage in the control and 20 µg/mL treatment in the third generation and the 40 µg/mL in the third and fourth generation groups differed significantly (Tukey, *p* < 0.001). The mean frequency of chromosomal changes observed in the third generation was 0.11 (SD = 0.15) in the 20 µg/mL, 0.28 (SD = 0.21) in the 40 µg/mL, and 0.09 (SD = 0.11) in the 60 µg/mL treatment groups; these values increased in the subsequent (fourth) generation to 0.20 (SD = 0.22), 0.30 (SD = 0.21), and 0.13 (SD = 0.21), respectively.

The oviposition of the female mosquitoes decreased significantly with increasing isodillapiole concentrations and in successive generations of exposure (ANOVA, *p* < 0.001). All treatments in all generations presented a reduction in oviposition compared to that in the negative control (Tukey test, *p* < 0.001), except for the 20 μg/mL treatment group in G_1_. The greatest variation occurred in the 60 μg/mL treatment group (Tukey, *p* < 0.001), with an average of 54.4 (SD = 7.3) eggs per mosquito pair in G_1_ and 18.1 (SD = 4.3) eggs per pair in G_4_. In the control group, the mean number of eggs per pair did not vary significantly (Tukey test: *p* < 0.05), with a mean of 89.8 (SD = 15.7) eggs per pair in G_1_ and 94.9 (SD = 11.7) in G_4_ (Table 1).

**TABLE 1:** Mean number of eggs laid per *Aedes aegypti* female exposed to three concentrations of isodillapiole and in the negative control group (0 µg/mL) over four consecutive generations (G1 to G4).

Isodillapiole (µg/mL)	Generation			
	G1	G2	G3	G4
0	89.8 (SD 15.7)	103.3 (SD 12.7)	101.7 (SD 17.1)	94.9 (SD 11.7)
20	78.4 (SD 6.9)	60.1 (SD 11.7)	56.8 (SD 7.8)	42.6 (SD 15.5)
40	63.0 (SD 10.4)	45.8 (SD 11.1)	47.0 (SD 17.4)	36.4 (SD 13.9)
60	54.4 (SD 7.3)	36.6 (SD 13.0)	29.6 (SD 12.6)	18.1 (SD 4.3)

**SD:** standard deviation.

## DISCUSSION

The semi-synthetic compounds derived from *P. arboreum*, *P. marginatum*, and *P. aduncum* belong to the phenylpropanoid group, which includes apiol, myristicin, eugenol, safrole, phenylpropanoid dimers, and dillapiole^21^. Dillapiole causes toxicity in *Drosophila melanogaster, Ae. atropalpus* ^22^, and *Ae. aegypti* ^8^. However, isodillapiole semi-synthetic has unknow effect on the control of insects, but our results may be a potential alternative for controlling *Ae. aegypti*, linked to more future field research to optimize our data.

The results of this study, regarding the effects of isodillapiole, confirm the reduction in oviposition when *Ae. aegypti* are exposed to dillapiole (200 and 400 μg/mL)^8^, which requires much higher concentrations than those of the isodillapiole assayed here. Isodillapiole reduced the number of hatched eggs laid by *Ae. aegypti*, with the greatest reduction being recorded in generations G_3_ and G_4_ at a concentration of 60 µg/mL. 

A reduction in egg production in the adults has also been observed when larvae are exposed to other semi-synthetic dillapiole compounds, such as ethyl ether dillapiole (50, 70, and 80 µg/mL) and *n*-butyl ether dillapiole (20, 25, and 30 µg/mL)^14^. Although the present study evaluated the effects of only isodillapiole on oviposition in *Ae. aegypti*, ethyl ether dillapiole (50 and 70 µg/mL) and *n*-butyl ether dillapiole (12.5 and 20 µg/mL) had similar effects on oviposition in *Ae. albopictus* in a previous study, which indicates the potential for the use of isodillapiole for the control of this species as well^15^.

The genotoxic effects of isodillapiole were evident from the analysis of interphasic nuclei and cells in metaphase, which presented abnormalities, such as micronuclei, budding, polynucleated cells, and anaphasic bridges. The frequency of anomalies increased (also observed in the later generations, G3 and G4 as well) when higher isodillapiole concentrations were used, indicating a dose-dependent effect, which further indicates a cumulative effect in the *Ae. aegypti* cells. Increased abnormalities in the *Ae. aegypti* cells indicate the genotoxic potential of this substance, corroborating previous results of assays, in which *Ae. aegypti* were exposed to dillapiole^8^ and semi-synthetic compounds of dillapiole^14^ ^,^ ^23^ ^,^ ^24^.

The results of the present analysis of isodillapiole suggest its genotoxic potential at much lower concentrations than those of dillapiole (200 and 400 μg/mL)^8^. Higher concentrations of other semi-synthetic compounds, such as ethyl ether dillapiole (at concentrations of 50, 70, and 80 µg/mL) and *n-*butyl ether dillapiole (at 20, 25, and 30 µg/mL)^14^, were needed to induce genotoxic effects in *Ae. aegypti*. Ethyl ether dillapiole at concentrations of 50 and 70 µg/mL, and *n*-butyl ether dillapiole at 12.5 and 20 µg/mL were also tested in *Ae. albopictus*, and produced similar results^15^.

Isodillapiole caused mortality in *Ae. aegypti* at all concentrations and presented genotoxic effects on oviposition patterns. Although severe greater effects have been reported in previous studies of other semi-synthetic compounds in *in vitro* assays of *Aedes* species, as in the present study, it is necessary to evaluate the efficacy of these compounds in more natural environmental settings. Additionally, the evaluation of the effects of exposure in non-target species to allow the optimal selection of these chemicals as an alternative approach must be performed for the control of populations of this mosquito.

In conclusion, isodillapiole showed greater toxic and genotoxic effects on *Ae. aegypti* (increased frequency of nuclear and chromosomal changes, decreased oviposition rates) compared to those of dillapiole, although much lower concentrations were required to provoke the same effects. Cellular damage and the reduction in oviposition rates were greatest at the highest concentration in the last generation, which indicated dose-dependent and cumulative effects.

## References

[1] Kurane I (2007). Dengue hemorrhagic fever with special emphasis on
immunopathogenesis. Comp Immunol Microbiol Infect Dis.

[2] Kraemer MUG, Sinka ME, Duda KA, Mylne AQN, Shearer FM, Barker CM (2015). The global distribution of the arbovirus vectors Aedes aegypti
and Aedes albopictus. Elife.

[3] Kindhauser MK, Allen T, Frank V, Santhana RS, Dye C (2016). Zika: the origin and spread of a mosquitos-borne
virus. Bull World Health Organ.

[4] Adler PH, Moncada-Álvarez LI (2016). Entomología médica, una necesidad. Rev Salud Publica.

[5] (2020). Epidemiological Update: Dengue. Epidemiological Update Dengue.

[6] Valle D, Bellinato DF, Viana-Medeiros PF, Lima JBP, Martins-Junior ADJ (2019). Resistance to temephos and deltamethrin in Aedes aegypti from
Brazil between 1985 and 2017. Mem Inst Oswaldo Cruz.

[7] Pohlit AM, Quignard ELJ, Nunomura SM, Tadei WP, Hidalgo ADF, Pinto ACS (2004). Screening of plants found in the State of
Amazonas. Acta Amazon.

[8] Rafael MS, Hereira-Rojas WJ, Roper JJ, Nunomura SM, Tadei WP (2008). Potential control of Aedes aegypti (Diptera: Culicidae) with
Piper aduncum L. (Piperaceae) extracts demonstrated by chromosomal
biomarkers and toxic effects on interphase nuclei. Genet Mol Res.

[9] Pandiyan GN, Mathew N, Munusamy S (2019). Larvicidal activity of selected essential oil in synergized
combinations against Aedes aegypti. Ecotoxicol Environ Saf.

[10] Maia JGS, Silva ML, Luz AIR, Zoghbi MGB, Ramos LS (1987). Espécies de Piper da Amazônia ricas em safrol. Quim Nova.

[11] Brazão MAB, Brazão FV, Maia JGS, Monteiro MC (2014). Antibacterial activity of the Piper aduncum oil and dillapiole,
its main constituent, against multidrug-resistant strains. Bol Latinoam Caribe Plantas Med Aromat.

[12] Ferreira R, Monteiro M, Silva J, Maia J (2016). Antifungal. Action of the Dillapiole-rich Oil of Piper aduncum
against Dermatomycoses Caused by Filamentous Fungi. Br J Med Med Res.

[13] Pinto ACS, Nogueira KL, Chaves FCM, Silva LVS, Tadei WP PA, Pohlit AM (2012). Adulticidal activity of dillapiol and semi-synthetic derivatives
of dillapiol against Aedes aegypti (L) (Culicidae). J Mosq Res.

[14] Domingos PRC, da Silva Pinto AC, dos Santos JMM, Rafael MS (2014). Insecticidal and genotoxic potential of two semi-synthetic
derivatives of dillapiole for the control of Aedes (Stegomyia) aegypti
(Diptera: Culicidae). Mutat Res Genet Toxicol Environ Mutagen.

[15] Meireles SF, Domingos PRC, Pinto ACS, Rafael MS (2016). Toxic effect and genotoxicity of the semisynthetic derivatives
dillapiole ethyl ether and dillapiole n-butyl ether for control of Aedes
albopictus (Diptera: Culicidae). Mutat Res Genet Toxicol Environ Mutagen.

[16] Akstein E (1962). The chromosomes of Aedes aegypti, and of some other
mosquitos. Bull Res Counc Isr.

[17] Pinto ACS (2008). Desenvolvimento de substâncias semissintéticas e bioativas, a partir de
4-nerolidilcatecol e dilapiol.

[18] Consoli RAGB, Oliveira RL (1994). Mosquitos de importância sanitária do Brasil.

[19] Imai HT, Taylor RW, Crosland MWJ, Crozier RH (1988). Modes of spontaneous chromosomal mutation and karyotype evolution
in ants with reference to the minimum interaction hypothesis. Japanese J Genet.

[20] Rafael MS, Tadei WP (1998). Metaphase karyotypes of Anopheles (Nyssorhynchus) darlingi Root
and Anopheles (Nyssorhynchus) nuneztovari Galbadón (Diptera:
Culicidae). Genet Mol Biol.

[21] Costa JGM, Santos PF, Brito SA, Rodrigues FFG, Coutinho HDM (2010). Composição Química e Toxicidade de Óleos Essenciais de Espécies
de Piper Frente a Larvas de Aedes aegypti L. (Diptera :
Culicidae). Lat Am J Pharm.

[22] Belzile AS, Majerus SL, Podeszfinski C, Guillet G, Durst T, Arnason JT (2000). Dillapiol derivatives as synergists: Structure-activity
relationship analysis. Pestic Biochem Physiol.

[23] Lima VS, Pinto AC, Rafael MS (2015). Effect of isodillapiole on the expression of the insecticide
resistance genes GSTE7 and CYP6N12 in Aedes aegypti from central
Amazonia. Genet Mol Res.

[24] Silva JS, Pinto ACS, Santos LHF, Rafael MS, Salgado Yvanna Carla de Souza (2019). Efeito ovicida e larvicida do éter metil dilapiol (EMD) em Aedes
aegypti, Manaus-AM. Patologias: Doenças Parasitárias.

